# HR-MRI-based nomogram network calculator to predict stroke recurrence in high-risk non-disabling ischemic cerebrovascular events patients

**DOI:** 10.3389/fneur.2024.1407516

**Published:** 2024-07-03

**Authors:** Zi-ang Li, Yu Gao, Lin Han, Bei-chen Xie, Yan-cong Sun, Xiao-yang Zhai, Ping Zhang, Yong-dong Li, Jun-yan Yue, Rui-fang Yan, Hong-Kai Cui

**Affiliations:** ^1^Department of Radiology Center, The First Affiliated Hospital of Xinxiang Medical University, Xinxiang, China; ^2^Department of Neurology Center, The First Affiliated Hospital of Xinxiang Medical University, Xinxiang, China; ^3^Institute of Diagnostic and Interventional Radiology, Shanghai Sixth People's Hospital Affiliated to Shanghai Jiao Tong University School of Medicine, Shanghai, China; ^4^Department of Neurointerventional Center, The First Affiliated Hospital of Xinxiang Medical University, Xinxiang, China

**Keywords:** stroke recurrence, high-resolution vessel wall imaging, nomogram, plaque, high-risk non-disabling ischemic cerebrovascular events

## Abstract

**Background and objective:**

To investigate the use of high-resolution magnetic resonance imaging (HR-MRI) to identify the characteristics of culprit plaques in intracranial arteries, and to evaluate the predictive value of the characteristics of culprit plaques combined with the modified Essen score for the recurrence risk of high-risk non-disabling ischemic cerebrovascular events (HR-NICE) patients.

**Methods:**

A retrospective analysis was conducted on 180 patients with HR-NICE at the First Affiliated Hospital of Xinxiang Medical University, including 128 patients with no recurrence (non-recurrence group) and 52 patients with recurrence (recurrence group). A total of 65 patients with HR-NICE were collected from the Sixth Affiliated Hospital of Shanghai Jiaotong University as a validation group, and their modified Essen scores, high-resolution magnetic resonance vessel wall images, and clinical data were collected. The culprit plaques were analyzed using VesselExplorer2 software. Univariate and multivariate logistic regression analyses were used to identify independent risk factors for recurrence, and a nomogram was constructed using R software to evaluate the discrimination of the model. The area under the curve (AUC) of the receiver operating characteristic curve (ROC) was used to evaluate the model performance. Calibration curves and Decision Curve Analysis (DCA) were used to evaluate the model efficacy.

**Results:**

Intra-plaque hemorrhage (OR = 3.592, 95% CI = 1.474–9.104, *p* = 0.006), homocysteine (OR = 1.098, 95% CI = 1.025–1.179, *p* = 0.007), and normalized wall index (OR = 1.114, 95% CI = 1.027–1.222, *p* = 0.015) were significantly higher in the recurrent stroke group than in the non-recurrent stroke group, and were independent risk factors for recurrent stroke. The performance of the nomogram model (AUC = 0.830, 95% CI: 0.769–0.891; PR-AUC = 0.628) was better than that of the modified Essen scoring model (AUC = 0.660, 95% CI: 0.583–0.738) and the independent risk factor combination model (AUC = 0.827, 95% CI: 0.765–0.889). The nomogram model still had good model performance in the validation group (AUC = 0.785, 95% CI: 0.671–0.899), with a well-fitting calibration curve and a DCA curve indicating good net benefit efficacy for patients.

**Conclusion:**

High-resolution vessel wall imaging combined with a modified Essen score can effectively assess the recurrence risk of HR-NICE patients, and the nomogram model can provide a reference for identifying high-risk populations with good clinical application prospects.

## 1 Introduction

China has one of the highest numbers of stroke patients globally, with stroke being the leading cause of death and disability among adults (1). Up to 80% of ischemic stroke patients may initially present with transient mild symptoms, like minor ischemic stroke and transient ischemic attack, without residual disability. However, many of these patients face stroke recurrence or progression due to unstable conditions, leading to severe clinical outcomes. This situation is termed high-risk non-disabling ischemic cerebrovascular events (HR-NICE). China has a large population of HR-NICE patients, and considering the national conditions and healthcare levels, they should be prioritized for cerebrovascular disease prevention and treatment (2). High-resolution magnetic resonance imaging (HR-MRI) has recently become crucial for assessing atherosclerosis. HR-MRI can identify various plaque components and pathological changes, providing a comprehensive evaluation of plaque vulnerability, stroke recurrence risk, and prognosis (3). The Essen Stroke Risk Score (ESRS) is one of the few effective tools for predicting recurrence risk in ischemic stroke patients. However, ESRS is relatively simple and does not incorporate significant risk factors like imaging features, limiting its predictive value. A prospective study in China showed that a modified ESRS, incorporating history of hypertension, diabetes mellitus, and TOAST classification of large artery atherosclerosis, better predicts recurrent cardiovascular events than the original ESRS. Yet, this modified score still has limitations, as it does not include critical risk factors like wall morphology and plaque characteristics prone to stroke recurrence (4). The nomogram, a data visualization tool, can visually display relationships between multiple variables, offering more comprehensive data analysis. It can directly calculate and show each variable’s contribution to outcomes, aiding in evaluating their importance and influence. This study aims to identify independent imaging risk factors associated with stroke recurrence using HR-MRI and construct a nomogram model that combines these factors with the modified Essen score. This will provide a more convenient and efficient reference for predicting stroke recurrence risk in HR-NICE patients.

## 2 Materials and methods

### 2.1 General information

Retrospectively collected data from patients with HR-NICE who were treated at the First Affiliated Hospital of Xinxiang Medical University from January 2020 to December 2022 as the training group, and retrospectively collected data from patients with HR-NICE who were treated at the Sixth Affiliated Hospital of Shanghai Jiaotong University from January 2022 to December 2022 as the validation group. The inclusion criteria were: (1) HR-NICE patients who met the diagnostic criteria of the “Guidelines for the Diagnosis and Treatment of High-Risk Non-Disabling Ischemic Cerebrovascular Events” (2); (2) Age ≥ 18 years old; (3) MRA, CTA or digital subtraction angiography has confirmed the presence of at least one stenosis (≥30%) in the intracranial artery that controls the symptomatic limb; (4) At least one risk of atherosclerosis factors, such as hypertension, diabetes, dyslipidemia, smoking, etc.; (5) The patient has completed the HR-MRI examination, the image is clear, and the clinical data required for this study are complete. Exclusion criteria: (1) Non-atherosclerotic vascular diseases, such as moyamoya disease or vasculitis; (2) Obvious moderate to severe disease in cervical arteries (common carotid artery, extracranial segment of internal carotid artery or vertebral artery) Patients with stenosis (stenosis >50%); (3) Patients with cardiogenic or hemorrhagic stroke; (4) HR-MRI examination image quality is poor or cannot tolerate magnetic resonance examination; (5) Patients’ clinical data are not recorded whole. Follow-up visits were conducted on the included patients for 1 year to determine whether they had a recurrence of ischemic cerebrovascular events. This study complies with the Helsinki Declaration and has obtained ethical approval from the two hospitals mentioned above, exempting participants from informed consent.

### 2.2 Imaging method

All patients were examined using a 16-channel combined head and neck coil and 3.0 T magnetic resonance equipment. Each patient first underwent diffusion-weighted imaging (DWI), three-dimension time-of-flight magnetic resonance angiography (3D-TOF MRA), T1-weighted imaging, T2-weighted imaging and T2 fluid attenuated inversion recovery (T2 Flair). After that, a 3D HR-MRI scan was performed. After the imaging was completed, gadobutrol (Gadavist, Bayer, 0.1 mmol/kg) was injected, followed by another 3D HR-MRI scan. The scanning parameters for each sequence are shown in Supplementary Table 1.

### 2.3 Image post-processing and analysis

All images were analyzed by two senior neuroimaging experts according to the patient’s clinical presentation, 3D-TOF MRA, and DWI results. Each detected plaque was classified as a “culprit” or “non-culprit “plaque. The definition of culprit plaque was the lesion appearing on the same side of the fresh infarction on the DWI image. If there were more than one plaque in the same vessel distribution area, the narrowest lesion was selected for analysis. The collected 3D HR-MRI data were normalized and reconstructed using VesselExplorer2 (Qingying Huakang Technology Co., Ltd., Beijing) post-processing workstation according to the American Society of Neuroradiology Vessel Wall Imaging Guidelines (5). Images were obtained in coronal, sagittal, and axial views to better display the state of plaques and vessel walls, and image quality was scored (1 point for poor image quality affecting observation; 2 points for most of the image being clear with only a small portion of the vessel wall being slightly blurred; 3 points for clear vessel wall outline). After excluding images with a quality score of 1, the software was used to analyze plaque characteristics, including plaque identification and delineation, intra-plaque hemorrhage (IPH), plaque enhancement, and stenosis rate. First, HR-MRI data were imported into the post-processing software and images were reconstructed perpendicular to the long axis of the vessel where the culprit plaque was located. The section where the culprit plaque was located was enlarged by 400%, and the outer contour of the vessel wall and intraluminal contour were manually delineated. The software automatically measured the corresponding vessel area, luminal area, and maximum wall thickness. The reference level of vessel area (VA) and lumen area (LA) preferentially selects the level of no significant stenosis at the proximal end of the corresponding lumen, followed by the level of no significant stenosis at the distal end of the corresponding lumen. The degree of vessel stenosis is calculated using the following method: stenosis rate = (1-LAmin/LAreference) × 100%; wall area (WA) = VA-LA; plaque area (PA) = WAmin-WAreference; remodeling index (RI) = VAmin/VAreference, RI ≥ 1.05 is positive remodeling, RI ≤ 0.95 is negative remodeling. Normalized wall index (NWI) = WA/VA. The stenosis rate less than 50% is grade 0, the stenosis rate between 50 and 69% is grade 1, and the stenosis rate between 70 and 99% is grade 2. Based on the signal intensity of the pituitary in the enhanced T1WI image, the plaque signal remains unchanged in grade 0; there is enhancement, but the degree of enhancement is lower than that of the pituitary enhancement in grade 1; the plaque enhancement degree is similar to that of normal pituitary enhancement is grade 2. Intraplaque hemorrhage (IPH) is defined as a T1WI signal higher than 150% of the adjacent muscle tissue signal (6–9).

### 2.4 Statistical analysis

SPSS 27.0 software was used for statistical analysis. Measurement data that were normally distributed were expressed as mean ± standard deviation ($\bar{x}$ ± s). Two independent sample t-tests were used for comparison. Measurement data that did not conform to normal distribution were expressed as median [M (Q25, Q75)], the Mann–Whitney U test was used for comparison, the count data was expressed as the number of cases [*n* (%)], and the chi-square test or Fisher’s exact probability method was used for comparison. Single-factor and multi-factor Logistics regression analysis were used to screen out independent risk factors affecting stroke recurrence, R software (version 4.3.1) was used to draw a nomogram, using ROC curves and Precision-Recall (PR) curve to analyze the model’s ability, drawing calibration curves to evaluate the consistency of the model, and using DCA curves to evaluate the clinical efficacy of the nomogram model, *p* < 0.05 was considered statistically significant.

## 3 Results

### 3.1 Clinical characteristics of patients in the training group

The patients in this study with an average age of (58.1 ± 10.8) years. Among them, 52 (28.89%) patients had stroke recurrence and 128 (71.11%) patients did not have stroke recurrence. Age, gender, BMI, history of atrial fibrillation, history of myocardial infarction, history of diabetes, history of hypertension, history of cerebrovascular disease, history of alcohol consumption, admission NIHSS score, total cholesterol, triglycerides, and low-density lipoprotein of patients in the recurrence group and the non-recurrence group There was no statistical significance in protein cholesterol, high-density lipoprotein cholesterol, apolipoprotein A, apolipoprotein B, fibrinogen, blood glucose, and D-dimer (*p* > 0.05). There were statistically significant differences in smoking history and homocysteine (Hcy) between the two groups of patients (*p* < 0.05; Table 1).

**Table 1:** Clinical characteristics and imaging data of the non-recurrent group and recurrence group in the training group.

project	Non-recurrence group (*n* = 128)	Recurrence group (*n* = 52)	t/Z/χ^2^ value	*p*-value
Age [years, M (Q25, Q75)]	60 (51,67)	59 (50,66)	0.567	0.572
Male[*n*(%)]	85 (66.406)	37 (71.154)	0.382	0.537
BMI[kg/m^2^, M(Q25,Q75)]	24.1 (22.4,27.0)	25.3 (22.9,27.2)	−0.911	0.363
Smoking history[*n*(%)]	44 (34.375)	27 (51.923)	4.767	0.029
Drinking history[*n*(%)]	30 (23.438)	18 (34.615)	2.363	0.124
Hypertension [*n*(%)]	70 (54.688)	24 (46.154)	1.079	0.299
Diabetes[*n*(%)]	25 (19.531)	12 (23.077)	0.285	0.594
Previous myocardial infarction [*n*(%)]	7 (5.469)	4 (7.692)	0.319	0.572
Admission NIHSS score [M (Q25, Q75)]	1 (0,4)	2 (0,4)	−0.881	0.355
Total cholesterol [mmol/L, M(Q25, Q75)]	3.6 (3.1,4.6)	4.0 (3.4,4.5)	−1.329	0.184
Triglycerides [mmol/L, M(Q25, Q75)]	1.2 (0.9,1.6)	1.3 (0.9,2.1)	−1.225	0.221
LDL[mmol/L, M(Q25, Q75)]	2.2 (1.7,2.9)	2.4 (2.0,2.8)	−1.043	0.298
HDL[mmol/L, M(Q25, Q75)]	0.9 (0.8,1.2)	1.0 (0.8,1.2)	0.642	0.522
Apo A [mmol/L, M(Q25, Q75)]	1.1 (1.0,1.3)	1.1 (1.0,1.3)	−0.727	0.468
Apo B [mmol/L, M(Q25, Q75)]	0.8 (0.7,1.0)	0.9 (0.7,1.0)	−1.270	0.204
Fibrinogen [mg/dL, M(Q25, Q75)]	284 (256,331)	295 (257,345)	−0.858	0.391
Blood sugar [mmol/L, M(Q25, Q75)]	5.2 (4.7,6.3)	5.3 (4.8,6.1)	−0.825	0.410
D-dimer [mg/L, M(Q25, Q75)]	0.6 (0.5,0.8)	0.7 (0.5,0.8)	−0.368	0.711
Homocysteine [mmol/L, M(Q25, Q75)]	16.1 (13.0,20.2)	19.9 (17.7,21.3)	−4.097	<0.001
**Imaging data**				
Anterior circulation [*n*(%)]	70 (54.688)	25 (48.077)	0.648	0.421
Maximum wall thickness [mm, M (Q25, Q75)]	1.5 (1.2,1.8)	1.6 (1.3,1.8)	−1.365	0.173
NWI [M(Q25, Q75)]	75.4 (68.5,81.3)	82.0 (80.8,83.2)	−5.889	<0.001
**Stenosis grade [*n*(%)]**				
Level 0	54 (42.188)	9 (17.308)	16.086	<0.001
Level 1	52 (40.625)	21 (40.385)		
Level 2	22 (17.188)	22 (42.308)		
**Enhancement level [*n*(%)]**				
Level 0	55 (42.969)	11 (21.154)	9.713	0.008
Level 1	52 (40.625)	24 (46.154)		
Level 2	21 (16.406)	17 (32.692)		
Intra-plaque hemorrhage [*n*(%)]	21 (16.406)	31 (59.615)	33.607	<0.001
Positive reconstruction [*n*(%)]	37 (28.906)	24 (46.154)	4.910	0.027

BMI, body mass index; LDL, low-density lipoprotein cholesterol; HDL, high-density lipoprotein cholesterol; NWI, normalized wall index.

### 3.2 Analysis of patients’ HR-MRI data in the training group

**Figure 1:**
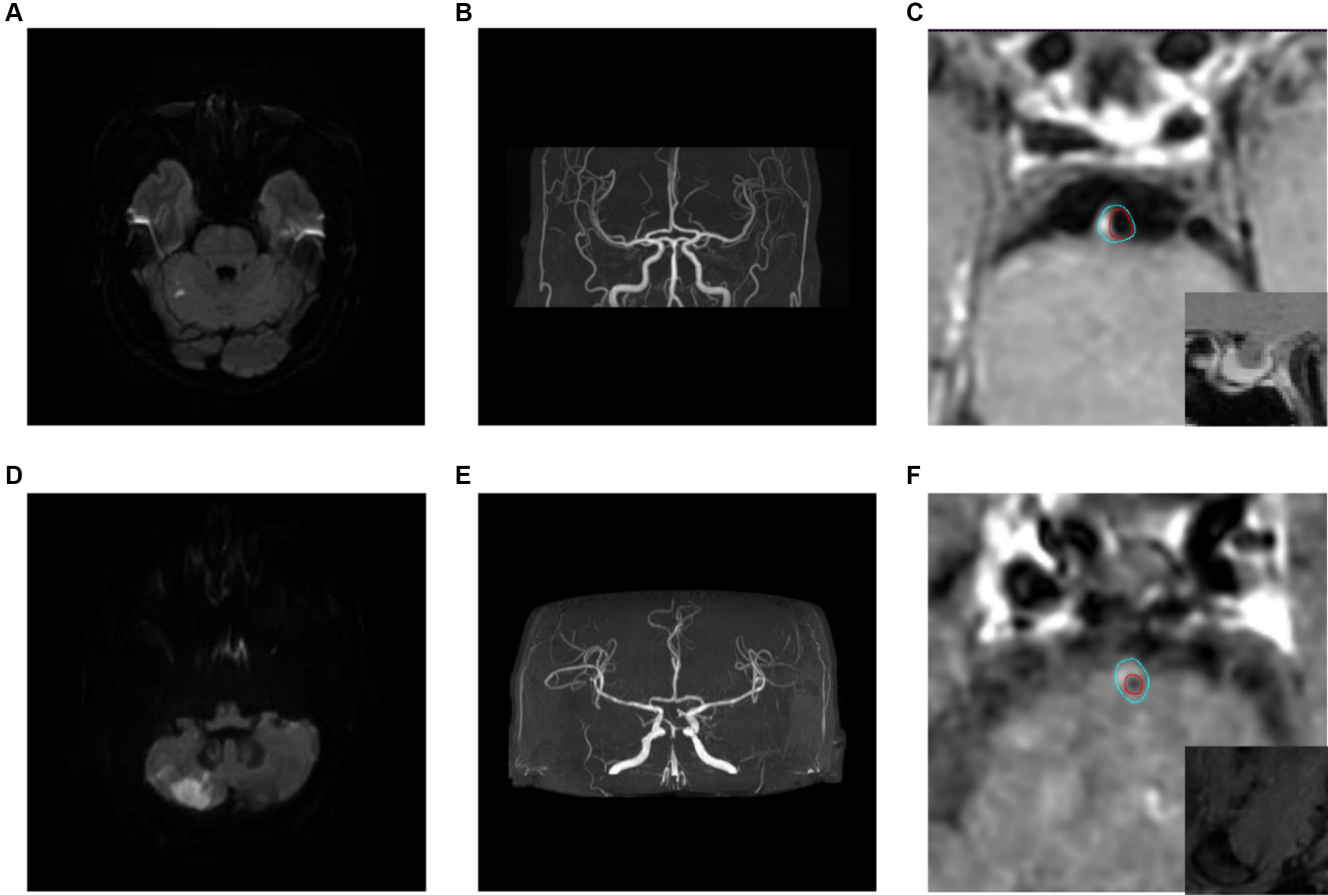
Typical HR-MRI images of patients in the recurrence group and non-recurrence group. **(A–C)** Male, 58 years old, with unsteady walking for 1 day. DWI showed acute cerebral infarction in the right cerebellar hemisphere **(A)**; MRA showed mild stenosis in the distal lumen of the basilar artery **(B)**; HR-MRI enhanced images show eccentric plaques in the basal artery with mild narrowing of the lumen, and the degree of plaque enhancement is higher than that of pituitary enhancement **(C)**; The blue line outlined the area of the wall, and the red line outlined the area of the lumen. **(D–F)** Male, 57 years old, with transient dizziness for more than 10 days. DWI showed acute cerebral infarction in the right cerebellar hemisphere **(D)**; MRA showed local severe stenosis in the basilar artery **(E)**; The HR-MRI image shows eccentric plaques in the basal artery, with severe narrowing of the lumen. The plaque signal is significantly higher than that of the adjacent medial pterygoid muscle, indicating the presence of Intra-plaque hemorrhage **(F)**.

There was no statistical significance in the plaque location distribution and maximum wall thickness between the recurrence group and the non-recurrence group (*p* > 0.05), but the plaque stenosis grade, plaque enhancement level, intra-plaque hemorrhage, positive reconstruction and normalized wall index were statistically significant (*p* < 0.05), see Table 1. Typical HR-MRI image data of patients in the recurrence group and non-recurrence group are shown in Figure 1.

### 3.3 Screening of risk factors for stroke recurrence and construction of combination models

**Figure 2:**
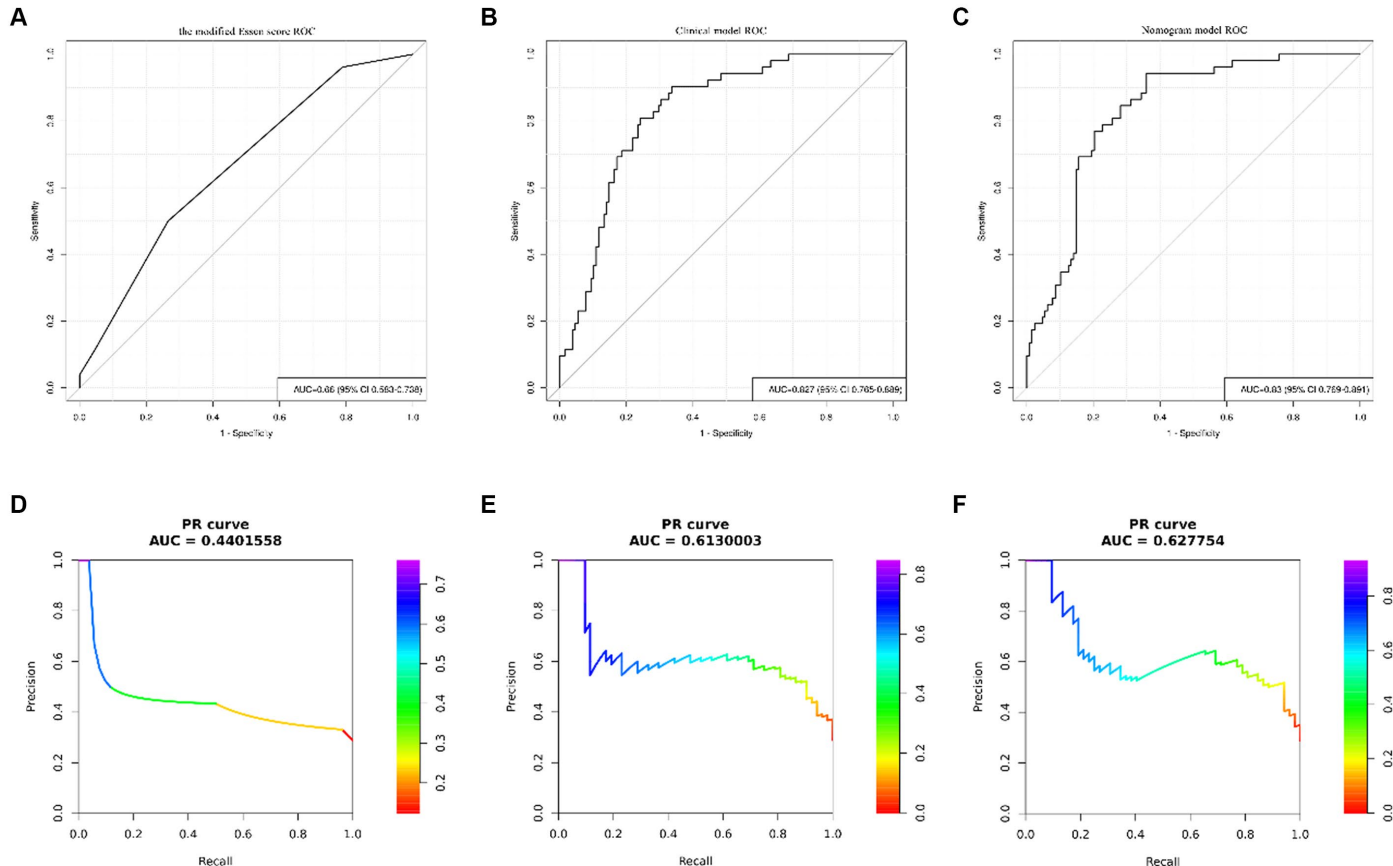
ROC curves and PR curves of different models in the training group. Panels **(A–C)** show the ROC curve of the modified Essen score, the ROC curve of the clinical model of independent risk factors screened by multivariate binary logistic regression, and the ROC curve of the nomogram model, respectively. Panels **(D–F)** show the PR curve of the modified Essen score, the PR curve of the clinical model of independent risk factors screened by multivariate binary logistic regression, and the PR curve of the nomogram model, respectively.

**Figure 3:**
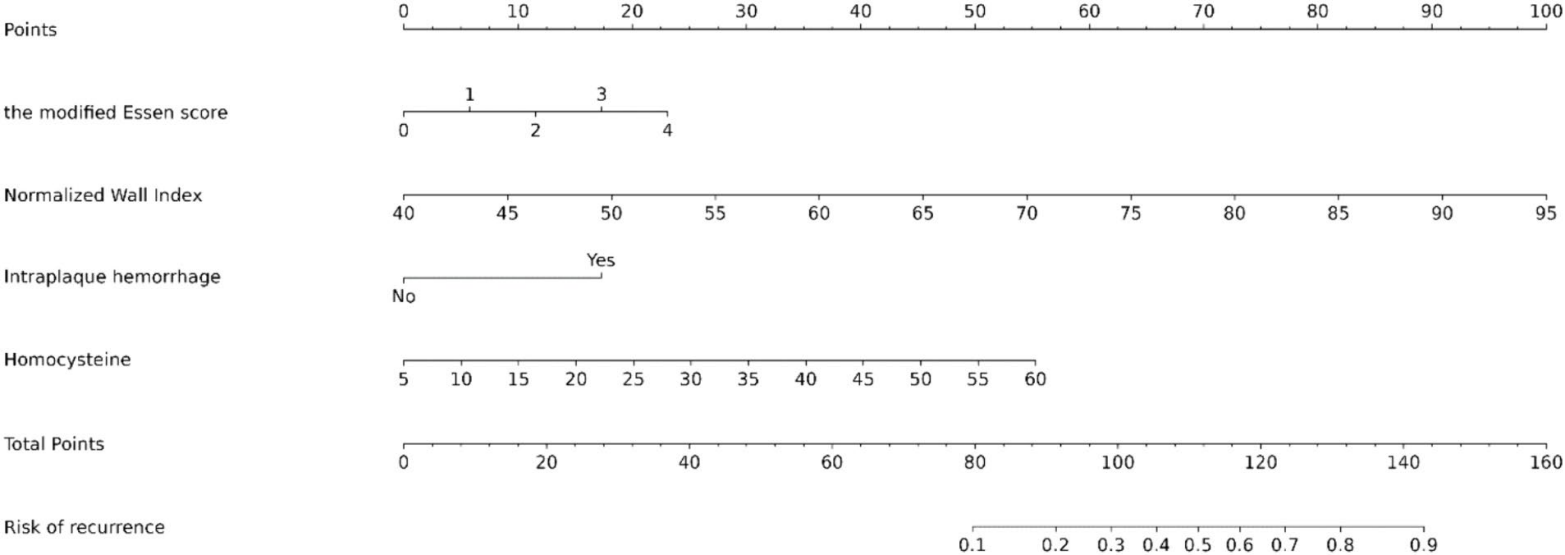
Nomogram predicting the risk of recurrence in HR-NICE patients. To obtain the probability of recurrent stroke risk for each HR-NICE patient, the “Total Points” is calculated by adding the respective “Points” values for each variable. Based on the corresponding total points, a vertical line can be drawn downward to obtain the probability of recurrence risk for each patient.

**Figure 4:**
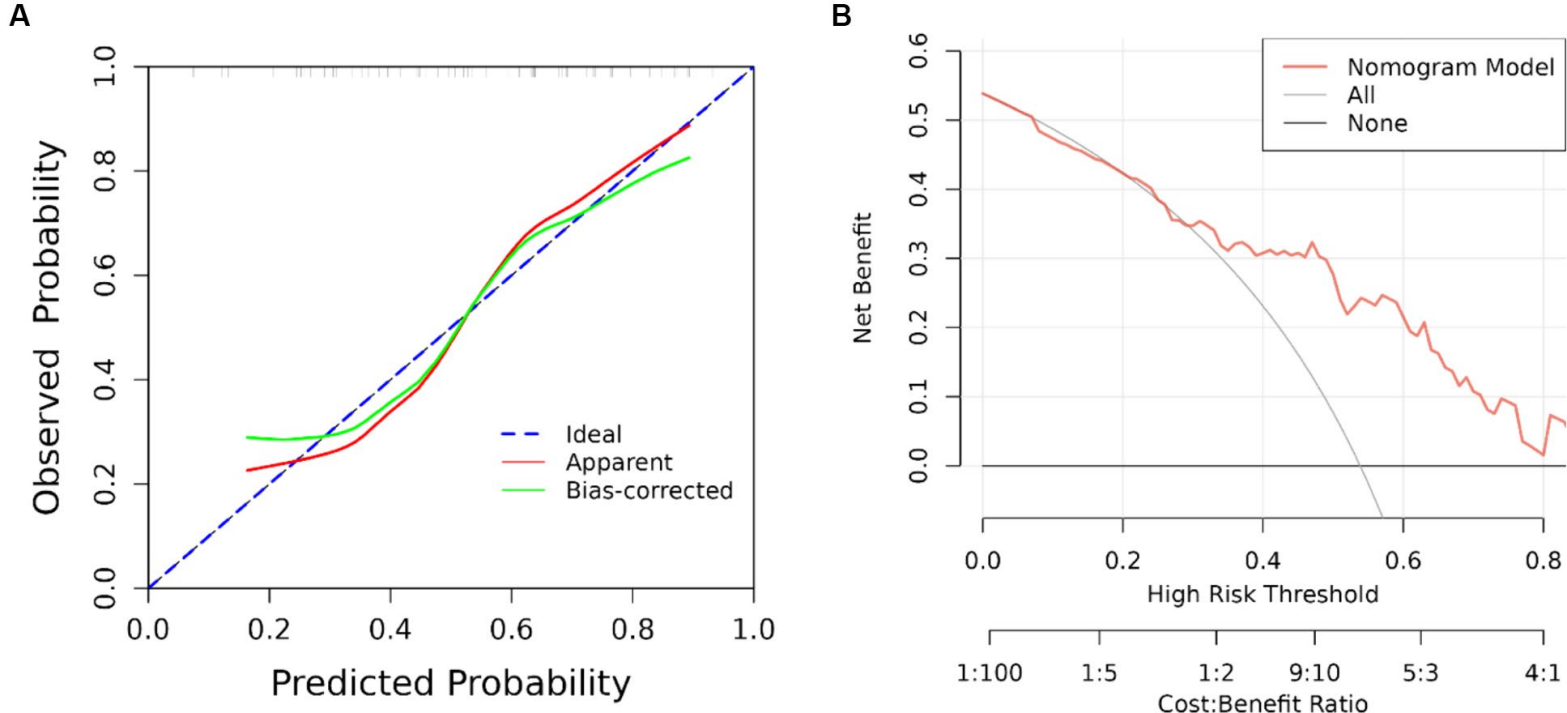
ROC curve, calibration curve and DCA curve of the validation group. **(A)** Calibration curve of the validation group and **(B)** clinical decision curve. The black straight line assumes all non-relapse patients, the gray curve assumes all relapse patients, and the red curve represents the nomogram model. The vertical axis is the net benefit rate, and the horizontal axis is the threshold probability.

The results of univariate analysis of all baseline data in the training group showed that normalized wall index, homocysteine, maximum wall thickness, stenosis grade, enhancement level, intraplaque hemorrhage, positive reconstruction, and smoking history were associated with recurrence of stroke in HR-NICE patients (Table 2). The risk factors with *p* < 0.1 in univariate analysis were included in multivariate binary logistic regression analysis. The results showed that normalized wall index (OR: 1.114; 95% CI: 1.027–1.222), homocysteine (OR: 1.098; 95% CI: 1.025–1.179), intra-plaque hemorrhage (OR: 3.592; 95% CI: 1.474–9.104) were independent risk factors for recurrence of stroke in HR-NICE patients (Table 2). Using the modified Essen score to construct a prediction model (AUC = 0.660, 95%CI: 0.583–0.738), using the independent risk factors screened in multivariate logistic regression analysis to construct a model (AUC = 0.827, 95%CI: 0.765–0.889), and using the modified Essen score and the risk factors in multivariate logistic regression to construct a prediction model (AUC = 0.830, 95%CI: 0.769–0.891) to predict the recurrence risk of HR-NICE patients. In our study, there was an imbalance between the stroke recurrence group and the non-recurrence group. When facing imbalanced datasets, PR-AUC better reflects the performance improvement of the model, so we evaluated the model using PR curves. The PR curve also showed that the combined prediction model constructed using the modified Essen score and the risk factors in multivariate logistic regression achieved good performance in the training group (PR-AUC = 0.627), as shown in Figure 2. Finally, we used R software to construct a nomogram model to predict the recurrence risk of HR-NICE patients by improving the Essen score, Normalized Wall Index, homocysteine, and intraplaque hemorrhage (Figure 3). The calibration curve showed that the nomogram model fitted well with the 45° diagonal line, indicating that the prediction of stroke recurrence probability by the nomogram model was highly consistent with the actual observed probability. The DCA curve showed that timely intervention using the nomogram model for HR-NICE patients could achieve good net benefits (Supplementary Figure 1). To demonstrate the better generalization of the model, we validated the constructed nomogram model in the validation group (AUC = 0.785, 95%CI: 0.671–0.899). The results showed that the model still had good diagnostic performance in the validation group. The calibration curve and DCA decision curve showed that the model could predict the recurrence risk of HR-NICE patients well (Figure 4).

**Table 2:** Logistic regression results of risk factors for recurrence group in HR-NICE patients in the training group.

Project	Univariate binary logistic regression			Multivariate binary logistic regression		
	OR	95%CI	*P*	OR	95%CI	*P*
NWI	1.193	1.112–1.28	0.000	1.114	1.027–1.222	0.015
Homocysteine	1.087	1.021–1.157	0.009	1.098	1.025–1.179	0.007
Maximum wall thickness	2.129	0.897–5.052	0.086			
**Stenosis grade**						
0						
1	2.423	1.016–5.777	0.046			
2	6.000	2.390–15.062	0.000			
Intra-plaque hemorrhage	7.522	3.643–15.530	0.000	3.592	1.474–9.104	0.006
Positive reconstruction	2.108	1.083–4.102	0.028			
**Enhancement level**						
0						
1	2.308	1.029–5.178	0.043			
2	4.048	1.629–10.055	0.003			
Smoking history	2.062	1.071–3.969	0.030			

NWI, Normalized Wall Index.

### 3.4 Online network calculator based on nomogram

**Figure 5:**
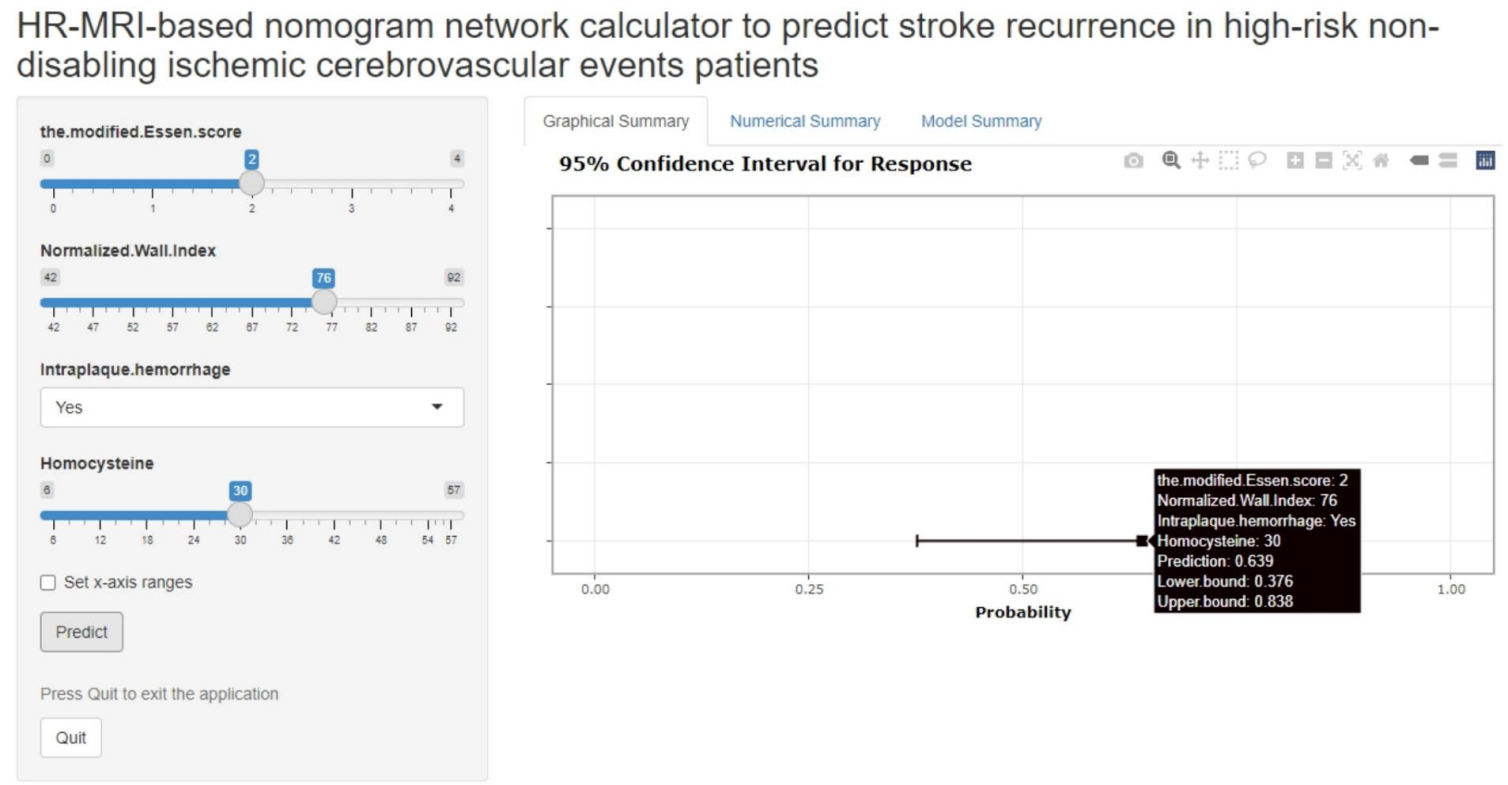
The picture above is a network calculator based on the nomogram model. The patient’s Normalized Wall Index is 76, Intra-plaque hemorrhage is present, the blood homocysteine level is 30 mmol/L, the modified Essen score is 2 points, and the prediction probability of the model is 0.639 (95%CI: 0.376–0.838).

The joint model represented by the nomogram has good performance in predicting the risk of recurrence in HR-NICE patients, so we have built an online network calculator based on the network^1^ (Figure 5) to facilitate the better use of this model in clinical practice.

## 4 Discussion

China is currently one of the countries with the highest burden of stroke, and for ischemic stroke, some patients can maintain a stable state for a long time after onset, with a good prognosis, but more patients are in a sub-stable or unstable state, which often has a high recurrence rate in a short period of time and may lead to adverse outcomes such as disability or death, seriously affecting the quality of life of patients. Related studies have shown that approximately 65% of patients with ischemic cerebrovascular diseases are of this type. HR-NICE patients have the highest risk of stroke recurrence at 3 months, and once recurrence occurs, it usually leads to further deterioration or even death of the remaining neurological function (10–12). Therefore, it is necessary to identify the recurrence factors of stroke in HR-NICE patients in a timely and effective manner, which can provide timely clinical intervention for patients with unstable conditions, reduce the risk of recurrence, and guide clinical prevention. This study used HR-MRI to conduct a more detailed study of the responsible plaques in intracranial arteries in HR-NICE patients. Through the evaluation of patient imaging data, we further screened the risk factors associated with ischemic event recurrence and combined them with the modified Essen score to perform timely and effective risk stratification for patients.

### 4.1 Correlation between clinical characteristics and stroke recurrence

Many previous studies have confirmed that serum homocysteine levels are related to the occurrence of various vascular events. The study by Del et al. (13) confirmed that the increase in Hcy levels after cerebral infarction is a predictor of recurrence of various vascular events. The increase in Hcy is often caused by a variety of factors, including insufficient intake or absorption of vitamin B6, lack of folic acid or vitamin B12, drug factors, and lifestyle factors (such as smoking, drinking, etc.) (14). The results of multiple animal experiments show that the increase in Hcy often leads to complex changes in the blood vessel wall, such as increased oxidative stress, pro-inflammatory effects, and endothelial dysfunction. This indicates that the increase in Hcy levels is related to the oxidative stress in the blood vessel wall, including pro-inflammatory effect and endothelial dysfunction (15, 16). The study by Holmen et al. (17) showed that when Hcy increases by five μmol/L, the risk of stroke increases by 43%. Therefore, we should pay more attention to the importance of Hcy in the examination of stroke patients. In the Chinese Adult Stroke Primary Prevention Trial (18), it was shown that patients in the folic acid treatment group had lower Hcy levels than the control group, and the probability of recurrent ischemic stroke was reduced by 24%. A review by Marti-Carvajal et al. (19) also found that patients receiving B12, folic acid, and B6 vitamins had a reduced risk of recurrent stroke compared with patients receiving placebo. This shows that for patients with high Hcy levels, timely and effective dietary control, can be of guiding significance in reducing the recurrence rate of ischemic cerebrovascular disease in the future. In addition, there was a statistical difference between smoking history and the recurrence of stroke events in univariate analysis. Smoking is an important risk factor for cardiovascular and cerebrovascular diseases, but we can avoid this risk factor by correcting our behavioral habits. Smoking causes endothelial dysfunction and atherosclerosis, including oxidative stress, reduced nitric oxide availability, increased monocyte adhesion, and the cytotoxic effects of nicotine, and increases coronary artery disease, myocardial infarction, and Risk of serious clinical complications such as stroke and peripheral arterial disease. Recent studies have also shown that there is a strong direct dose–response relationship between the number of cigarettes smoked and ischemic stroke. Although complete smoking cessation is a public health goal, reducing the number of cigarettes smoked can reduce the risk of ischemic stroke (20). There was no statistical difference in smoking history in the multivariate analysis. This may be due to the small sample size of this study, or it may be due to the fact that patients quit smoking promptly after experiencing TIA or minor stroke.

### 4.2 Correlation between plaque characteristics and stroke recurrence

The results of this study suggest that imaging characteristics of plaques, such as intraplaque hemorrhage and normalized wall index, are independent risk factors for recurrent ischemic cerebrovascular events in patients with HR-NICE. In recent years, studies have shown that the unstable characteristics of culprit plaques can predict the recurrence of future ischemic cerebrovascular events, and IPH has been proven to be a factor in plaque instability (21). In the process of plaque formation and organization, IPH is often caused by the unstable and immature vascular endothelium of new blood vessels, which causes the rupture of the capillary wall under the stimulation of hypoxia and inflammatory factors of the body, thus causing extravasation of blood (22). From a hemodynamic perspective, ruptured neovascular endothelium often leads to an increase in plaque volume and slope. The greater the slope of the lesion, the greater the longitudinal lumen curvature and the greater the stress. Larger slopes also disrupt local flow patterns, increasing wall shear stress and oscillatory shear index. The upstream plaque will bear the huge impact load caused by the accelerated blood flow, thus generating large wall shear stress. Larger wall shear stress may also exacerbate plaque vulnerability by accelerating endothelial dysfunction, weakening the plaque surface, and increasing the necrotic core (23). Physiologically and pathologically, IPH may destabilize atherosclerotic plaques by rapidly expanding the necrotic core and further promoting free cholesterol deposition through red blood cell membrane accumulation (21, 24). In addition, oxidative, proteolytic and inflammatory processes triggered by IPH, including leukocytes, platelets and plasma proteins, are also related to recurrent stroke events (25). The normalized wall index normalizes wall area to total vessel area and takes into account the inherent differences in wall area of vessels of different diameters, such as the basilar and middle cerebral arteries, providing a measure of lesion burden. In addition, considering that there are various ways of remodeling the lumen during plaque formation, simply measuring the stenosis area often cannot accurately reflect the stenosis of the responsible blood vessel, and NWI can well judge the current status of the blood vessel. A larger NWI often means that the patient has a greater plaque burden. An increase in the plaque burden may mean that the plaque becomes more stable, but it will also lead to the above-mentioned hemodynamic problems, thereby exacerbating the risk of plaque rupture and increasing the risk of plaque rupture. Leading to recurrence of stroke (26). The study by Ran et al. (27) showed that plaque burden is an independent risk factor for stroke recurrence in the middle cerebral artery blood supply area. For every 10% increase in plaque burden, the risk of stroke recurrence will increase by 2.26 times. The study by CX et al. also proved that when the patient’s culprit plaque has the same or close NWI, the occurrence of intra-plaque hemorrhage can often provide a more effective predictive value for stroke recurrence (28). However, the maximum wall thickness, stenosis grade, positive remodeling and enhancement grade that did not have statistical significance in the multiple binary logistic regression may be because the inclusion criteria of this study are the HR-NICE patient population, which are mostly TIA and mild cases. In patients with stroke, the clinical symptoms and imaging features may not be typical.

### 4.3 Correlation between modified Essen score and stroke recurrence

Individualized and customized prevention and treatment for HR-NICE patients often require timely prediction of potential stroke events before the patient occurs, which also relies on clinical data and predictive analysis. Choosing an appropriate and effective predictive score is an important tool for assessing patients’ risk of future ischemic cerebrovascular events, stratifying patients, and selecting preventive treatments. Chen et al. (29) prospectively collected data from a total of 3,316 ischemic stroke patients in multiple centers in China to evaluate the accuracy of ESRS in predicting stroke recurrence and combined vascular events in patients with different categories of ischemic stroke within 1 year. The results showed that the accuracy of ESRS in predicting stroke recurrence was approximately 0.63 (95% CI, 0.57 ± 0.69), demonstrating the ability of ESRS to risk stratify patients. Ling et al. (4) modified the ESRS based on the characteristics of stroke in the Chinese population by adding the duration of hypertension, duration of diabetes, and stroke subtypes classified by etiology to the ESRS and deleting evaluation factors that were not efficient in the Chinese population. The modified Essen score was obtained, which constitutes a 4-point clinical index scale and was confirmed in a prospective cohort study based on the Chinese population to have an excellent ability to predict recurrent ischemic stroke and cardiovascular events. It is superior to ESRS in terms of clinical practicability and ease of rapid assessment. Although the modified Essen score has shown higher accuracy in predicting recurrence, this scale still fails to incorporate imaging features that are prone to the recurrence of cerebrovascular events. Therefore, this study proposes to combine the improved Essen score with the imaging features provided by HR-MRI to further optimize the recurrence prediction performance of the HR-NICE patient population and guide clinicians to better risk stratify patients.

### 4.4 Innovation and clinical practicality of online network calculators

At present, nomograms have been widely used in various clinical prediction models. This study constructed nomograms using the selected independent risk factors to assist clinical doctors in further decision-making analysis. Considering that the use of nomograms is still quite cumbersome, we have further developed a network calculator, which is the first online network calculator designed for the HR-NICE patient population. In addition, doctors can still use network calculators to dynamically monitor the recurrence risk of patients and provide more accurate personalized treatment by recalculating their clinical indicators after corresponding treatment for high-risk patients.

This study has the following limitations. Firstly, although this study is a dual-center study, the sample size is still small, and the coverage of patients in different regions is limited. The results may be affected by different ethnic groups and lifestyle habits, and a more detailed classification of clinical risk factors for patients has not been achieved. In the future, more center and large sample size trials are needed to further improve. Secondly, this study is a retrospective study, and it is more reasonable to use prospective studies to study the risk of patient recurrence. Clinical data and HR-MRI imaging features can be collected in a timely manner when patients experience recurrence. In future research, we will prospectively recruit more HR-NICE patients from multiple centers and establish a database to include more comprehensive risk factors for stroke recurrence. We will further deepen the research of HR-MRI technology, extract imaging omics features of responsible plaques and vascular walls, and conduct more detailed imaging omics, machine learning, and deep learning, aiming to further improve the predictive efficiency of the risk of recurrent ischemic cerebrovascular disease in the HR-NICE patient population.

## 5 Conclusion

In summary, this study found that modified Essen score, homocysteine, intra-plaque hemorrhage and normalized wall index are independent risk factors for recurrent ischemic cerebrovascular events in HR-NICE patients. The nomogram constructed by combining the modified Essen score with clinical and imaging features can improve the prediction performance of recurrent ischemic cerebrovascular events in HR-NICE patients. Based on this nomogram, we have created an online network calculator to help doctors stratify the risk of HR-NICE patients and develop personalized treatment plans.

## Data availability statement

The raw data supporting the conclusions of this article will be made available by the authors, without undue reservation.

## Ethics statement

The studies involving humans were approved by the Ethics Committee of the First Affiliated Hospital of Xinxiang Medical University. The studies were conducted in accordance with the local legislation and institutional requirements. Written informed consent for participation was not required from the participants or the participants’ legal guardians/next of kin because this study complies with the Helsinki Declaration and has obtained ethical approval, exempting participants from informed consent.

## Author contributions

Z-aL: Conceptualization, Writing – original draft, Writing – review & editing. YG: Conceptualization, Methodology, Writing – review & editing, Writing – original draft. LH: Data curation, Writing – review & editing. B-cX: Data curation, Writing – review & editing. Y-cS: Data curation, Writing – review & editing. X-yZ: Software, Writing – review & editing. PZ: Methodology, Writing – review & editing. Y-dL: Methodology, Writing – review & editing. J-yY: Methodology, Writing – review & editing. R-fY: Methodology, Project administration, Writing – review & editing. H-KC: Project administration, Supervision, Writing – review & editing.

## Footnotes

^1^ https://lza864545601.shinyapps.io/dynnomapp/
