# Supplementary material for: HR-MRI-based nomogram network calculator to predict stroke recurrence in high-risk non-disabling ischemic cerebrovascular events patients
Source: Front Neurol. 2024 Jul 3;15:1407516. doi: 10.3389/fneur.2024.1407516 (PMC11252045; doi:10.3389/fneur.2024.1407516)
Supplement: Supplementary file 1 [file Data_Sheet_1.docx]

**STable 1** **Summery of imaging parameters**

**Machine1. PHILIPS Ingenia Elition 3.0T**

| **Sequence** | **HRMRI** | |  | **Head MRI** | | | |
| --- | --- | --- | --- | --- | --- | --- | --- |
|  | 3D T1WI | 3D-TOF MRA |  | Axial T1WI | Axial T2WI | DWI | T2 Flair |
| FOV (mm) | 250×161 | 200×158 |  | 230×189 | 230×230 | 230×230 | 230×190 |
| Matrix | 312 × 201 | 308×168 |  | 288×178 | 244×244 | 152×122 | 308×203 |
| Slice thickness(mm) | 0.6 | 1.2 |  | 5.0 | 5.0 | 5.0 | 5.0 |
| Resolution | 0.8×0.8×0.8 | 0.65×0.94×1.2 |  | 0.8×1.05 | 0.95×0.95 | 1.5×1.89 | 0.75×0.91 |
| TR/TE（ms） | 600/31 | 19/3.5 |  | 2373/20 | 2756/105 | 2194/86 | 8000/120 |
| TOF, time of flight; DWI, diffusion-weighted imaging; FLAIR, fluid-attenuated inversion recovery; FOV, field of view; TR, repetition time; TE, echo time. | | | | | | | |

**Machine2. SIEMENS MAGNETOM Vida**

| **Sequence** | **HRMRI** | |  | **Head MRI** | | | |
| --- | --- | --- | --- | --- | --- | --- | --- |
|  | 3D T1WI | 3D-TOF MRA |  | Axial T1WI | Axial T2WI | DWI | T2 Flair |
| FOV (mm) | 220×188 | 200×181 |  | 230×230 | 230×230 | 230×230 | 230×230 |
| Matrix | 220×188 | 200×181 |  | 230×230 | 230×207 | 230×230 | 230×184 |
| Slice thickness(mm) | 0.69 | 0.7 |  | 5.0 | 5.0 | 5.0 | 5.0 |
| Resolution | 0.7×0.7×0.7 | 0.3×0.3×0.7 |  | 0.8×0.8 | 0.4×0.4 | 0.7×0.7 | 0.8×0.8 |
| TR/TE（ms） | 900/28 | 21/3.42 |  | 2000/7.5 | 5000/92 | 5000/70 | 8000/97 |
| TOF, time of flight; DWI, diffusion-weighted imaging; FLAIR, fluid-attenuated inversion recovery; FOV, field of view; TR, repetition time; TE, echo time. | | | | | | | |

**SFigure 1** Calibration curve and DCA decision curve of the nomogram model in the training group


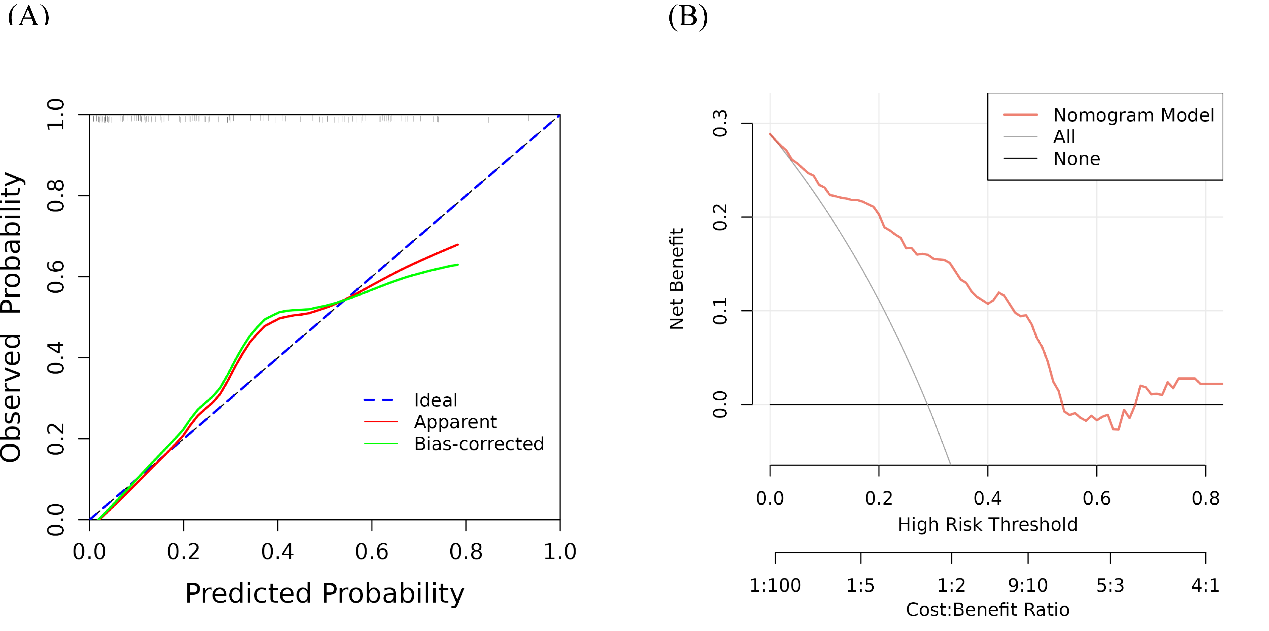


**SFigure 1.** Figure A is the calibration curve of the nomogram model in the training group, and Figure B is the DCA decision curve of the nomogram model in the training group.
